# Resistance and Impact Training During Weight Loss Improves Physical Function and Body Composition in Older Adults With Obesity

**DOI:** 10.1002/jcsm.13789

**Published:** 2025-04-09

**Authors:** Jakub Mesinovic, Anoohya Gandham, Mavil May Cervo, Paul Jansons, Costas Glavas, Michael Braude, Juan Pena Rodriguez, Barbora De Courten, Ayse Zengin, Belinda R. Beck, Peter R. Ebeling, David Scott

**Affiliations:** ^1^ Institute for Physical Activity and Nutrition (IPAN), School of Exercise and Nutrition Sciences Deakin University Burwood Victoria Australia; ^2^ Department of Medicine, School of Clinical Sciences at Monash Health Monash University Clayton Victoria Australia; ^3^ Mary MacKillop Institute for Health Research Australian Catholic University Fitzroy Victoria Australia; ^4^ School of Health and Biomedical Sciences RMIT University Melbourne Victoria Australia; ^5^ Menzies Health Institute Queensland Griffith University, Gold Coast campus Southport Queensland Australia; ^6^ Exercise Science, School of Health Sciences and Social Work Griffith University, Gold Coast campus Southport Queensland Australia; ^7^ The Bone Clinic Brisbane Queensland Australia

**Keywords:** aerobic training, bone, exercise, muscle, obesity, older adults, resistance and impact training, sarcopenia, weight loss

## Abstract

**Background:**

Weight loss achieved via energy restriction leads to significant losses in muscle and bone mass, potentially increasing risk for sarcopenia and osteoporosis. High‐intensity resistance and impact training (HiRIT) might attenuate weight loss–induced musculoskeletal declines. Our objective was to compare changes in physical function and body composition in older adults with obesity undertaking dietary weight loss combined with HiRIT or aerobic training (AT).

**Methods:**

Sixty older adults (aged ≥ 60 years) with obesity (dual‐energy x‐ray absorptiometry determined body fat percentage ≥ 30% in men and ≥ 40% in women) and a mobility limitation (Short Physical Performance Battery [SPPB] score ≤ 11) were randomly assigned to either 12 weeks of supervised, centre‐based HiRIT or self‐directed, home‐based AT while consuming a hypocaloric diet (750–1000 kcal/day reduction in energy intake). Changes in physical function (primary outcome: gait speed) and body composition were compared between groups.

**Results:**

A total of 49/60 randomised participants (mean age: 69.6 ± 6 years; 58% women; mean BMI: 32.9 ± 4.1 kg/m^2^) completed the trial. Gait speed increased following HiRIT compared with AT (mean difference: 0.07 m/s [95% CI: 0.01, 0.13]). Chair stand times decreased in both groups (HiRIT: −1.3 s [95% CI: −2.1, −0.4] vs. AT: −0.8 s [95% CI: −1.6, −0.04]) and HiRIT, but not AT, increased handgrip strength (HiRIT: 2.2 kg [95% CI: 0.6, 3.9] vs. AT: 0.7 kg [95% CI: −0.9, 2.3]) and SPPB scores (HiRIT: 0.9 [95% CI: 0.4, 1.3] vs. AT: 0.4 [95% CI: −0.04, 0.8]). Similar decreases in total body mass (HiRIT: −5.1 kg [95% CI: −6.7, −3.4] vs. AT: −4.9 kg [95% CI: −6.5, −3.3]), fat mass (HiRIT: −3.6 kg [95% CI: −5.0, −2.2] vs. AT: −3.3 kg [95% CI: −4.7, −2.0]), visceral fat (HiRIT: −32.1 cm^2^ [95% CI: −47.4, −16.8] vs. AT: −31.4 cm^2^ [95% CI: −46.1, −16.8]) and appendicular lean mass (HiRIT: −0.8 kg [95% CI: −1.4, −0.2] vs. AT: −1.2 kg [95% CI: −1.8, −0.6]) were observed. HiRIT was well tolerated with only seven minor adverse events compared with five reported in those who completed AT.

**Conclusion:**

HiRIT appears to be safe and more effective than AT for improving gait speed in older adults with obesity undertaking dietary weight loss. Additional trials with larger sample sizes and longer durations are warranted to explore whether HiRIT can attenuate weight loss–related muscle and bone mass declines. **Trial Registration:** Australian New Zealand Clinical Trials: ACTRN12618001146280.

## 1 Background

The prevalence of obesity in older adults is increasing [1]. Older adults with obesity have an increased risk for poor musculoskeletal health, resulting in high rates of disability [2]. Sarcopenia, the age‐related decline in skeletal muscle mass and function [3], is also associated with increased disability risk [4]. Older adults with sarcopenia and obesity (i.e. sarcopenic obesity) have an even greater risk of falls and fractures compared to counterparts who have obesity alone [5, 6]. Hypocaloric diets are the most effective intervention for treating obesity and poor metabolic health through fat loss [7]. However, they can result in concomitant declines in muscle and bone mass, potentially leaving individuals at greater risk of falls and fractures [8, 9].

Weight loss–related musculoskeletal declines can be attenuated with exercise, although this depends on the type of exercise performed [8, 9]. Generally, aerobic training (AT) accelerates musculoskeletal declines, whereas resistance training (RT) attenuates them, in older adults with obesity and mobility limitations undergoing dietary weight loss [10, 11, 12]. In 160 older adults with obesity undertaking weight loss, compared with AT, RT attenuated lean mass losses (RT: −2%; AT: −5%) and total hip bone density losses (RT: < −1%; AT: −2.6%), and led to greater muscle strength gains (RT: +19%; AT: −4%) [10]. Both exercise modes led to similar improvements in physical function scores [10]. The superiority of RT over AT for preserving musculoskeletal health during weight loss is largely related to the site‐specific mechanical loading achievable with RT [9, 13].

Although RT can improve musculoskeletal health, exercise prescription factors are important, particularly for muscle strength and bone health improvements. A recent meta‐analysis showed that high‐load RT leads to greater maximal strength improvements compared with low‐load RT [14]. Low‐load and low‐intensity RT has also been associated with smaller improvements in muscle strength, power and bone health compared with high‐intensity resistance and impact training (HiRIT) in postmenopausal women with low bone mass [15]. HiRIT improves the same musculoskeletal outcomes in middle‐aged and older men with low bone mass [16]. Despite these promising results, it remains unclear whether HiRIT is safe and effective for improving physical function, body composition and other measures of musculoskeletal health in older adults with obesity undertaking dietary weight loss.

Our primary aim for this study was to compare changes in physical function in older adults with obesity undertaking weight loss combined with HiRIT or AT. Our secondary aim was to compare changes in body composition between groups. We hypothesised HiRIT would improve physical function and body composition (i.e. increase fat mass losses and attenuate lean mass losses) compared with AT in this population.

## 2 Material and Methods

### 2.1 Study Design and Population

This 12‐week pilot randomised controlled trial (RCT) recruited 60 older adults (aged ≥ 60 years) with obesity (body fat percentage ≥ 30 [men] or ≥ 40 [women] determined by dual‐energy x‐ray absorptiometry [DXA]) [17] and a mobility impairment (Short Physical Performance Battery [SPPB] score of ≤ 11). This pragmatic approach was used to identify older adults with obesity at risk of sarcopenia (i.e. sarcopenic obesity) who were likely to benefit from a lifestyle intervention while also reducing ceiling effects observed with the SPPB test [18]. Exclusion criteria included inability to walk unassisted for 400 m in 15 min; non‐English‐speaking or having difficulty communicating with personnel due to hearing or speech problems; failure to score > 18 points out of 30 in the Mini‐Mental State Exam (MMSE) indicating moderate or severe cognitive impairment [19]; planning to be away > 4 weeks during the trial; currently residing in a nursing home; and participated in > 4 weeks of supervised exercise or dietary programme targeted for weight loss or strength gains in the last 6 months. Participants were also excluded if they self‐reported a diagnosis of progressive neurological disorders; schizophrenia or bipolar disorder; severe knee or hip osteoarthritis; cardiovascular disease; lung disease requiring regular use of corticosteroids or supplemental oxygen; renal disease requiring dialysis; hyper‐ or hypothyroidism; and any other disorder of such severity that life expectancy is less than 12 months. Participants were recruited through print and online recruitment methods from suburban communities in Melbourne, Australia, over 2 years (2019–2021). This RCT was completed at the Monash Health Translational Research Facility, Melbourne, Australia, conformed to CONSORT guidelines; was conducted according to the principles of the Declaration of Helsinki; was approved by the Monash Health Human Research Ethics Committee (Protocol ID: HREC/18/monH/399); and was prospectively registered with Australian New Zealand Clinical Trials Registry under ACTRN12618001146280. All participants provided written informed consent.

### 2.2 Blinding and Randomisation

The current study was a single‐blind RCT where investigators collecting outcome measures were blind to the exercise allocation of participants. As the trial involved exercise interventions, it was not possible to blind participants to their intervention arm allocation. Participants were randomised to either gym‐based HiRIT or home‐based AT after baseline testing by one member of the study team using computer‐generated block randomisation. The team member responsible for contacting participants and disclosing treatment allocations was not involved in participant recruitment or outcome assessment.

### 2.3 Intervention

During the 12‐week intervention period, all participants completed a dietary weight loss intervention, and equal numbers were randomised to either HiRIT or AT as described below.

#### 2.3.1 Dietary Intervention

All participants were prescribed a hypocaloric diet to facilitate weight loss. To determine the amount of energy in the diet prescribed to each participant, habitual dietary nutrient intake was assessed using a 3‐day food record form that was completed on two non‐consecutive weekdays and one weekend before the commencement of the study intervention and at the end of the intervention (12 weeks). To complete the food record, study participants listed all the foods and beverages they consumed throughout the day, including quantities. From these data, participant's diets were modified by deducting 750–1000 kcal/day from their habitual intake, aiming for an approximate 1.5–2.0 lb reduction in total body fat mass per week. Each participant was oriented by the study dietitian on how to quantify their intake using food models and measuring utensils. They were also educated on how to reduce food portion sizes and replace energy‐dense foods with those of lower energy but nutrient‐dense foods. Food records were validated by the dietitian through one‐on‐one interviews with the study participants at both time points.

Dietary nutrient intake of participants was processed using FoodWorks 8 Professional for Windows (Xyris Software (Australia), Pty Ltd.) containing the 2011–2013 Australian Food and Nutrient Database (AUSNUT) with 53 nutrient values for 5740 foods and beverages. The study dietitian conducted telephone interviews every week throughout the intervention to monitor and review dietary intakes and set behavioural goals. Participants with poor dietary compliance, as demonstrated by deviations from the diet prescription during the telephone interviews, were given a 7‐day dietary plan to improve compliance.

A protein (whey protein isolate; ~30 g per serve) and a combined vitamin D and calcium (Ostevit D + Ca plus; cholecalciferol: 1000 IU; calcium 600 mg) supplement were also provided to ensure adequate intakes of these important nutrients for musculoskeletal health were maintained while consuming hypocaloric diets. Participants were encouraged to consume these supplements daily, and compliance was monitored through weekly telephone interviews.

#### 2.3.2 HiRIT

The 12‐week gym‐based HiRIT intervention was modelled on the successful LIFTMOR trial [15] designed to reduce the risk of fractures in postmenopausal women. The HiRIT intervention in LIFTMOR led to improvements in bone density and all physical function outcomes assessed in the study including leg extensor and back extensor strength, timed up‐and‐go test times, five times sit‐to‐stand times, functional reach and lower‐limb neuromuscular performance, compared with a control group that performed twice‐weekly home‐based flexibility training combined with low‐load and low‐intensity RT (10–15 repetitions; < 60% 1‐repetition‐maximum [1RM]) [15]. LIFTMOR also reported high compliance to HiRIT (92%) and only one minor adverse event (AE) (a minor back strain) [15].

Participants allocated to HiRiT in the present study were prescribed a structured 12‐week, twice‐weekly, 30‐minute HiRIT programme. Each participant was encouraged to attend each session supervised by an accredited exercise physiologist at a local gymnasium (Healthwise Fitness; Monash Medical Centre, Clayton). Some exercise sessions were performed one‐on‐one, whereas others were performed in small groups of up to 2–3 participants. Attendance was recorded via an exercise sheet. All exercises were individually tailored, considering initial fitness, injuries or illness. The prescribed exercise programme used an Olympic bar, dumbbells and/or weight plates. All participants were individually prescribed four fundamental exercises (deadlift, overhead press, back squat and modified jumping chin‐ups) throughout the intervention period. To ensure a safe commencement of HiRIT exercise, the first 2 weeks of the intervention involved body weight only or low‐load exercise variants, with a focus on learning the movement patterns. Participants performed up to two sets of five repetitions of all four exercises at 50% of 1RM to serve as a warm‐up at each session, followed by five sets of five repetitions at an intensity of > 80%–85% 1RM. Loads of the four prescribed exercises were progressively increased to ensure they maintained the desired training intensity throughout the study. During each session, investigators filled in an exercise diary to monitor participants' progression throughout the exercise intervention.

#### 2.3.3 AT

The AT group was assigned a moderate‐intensity, home‐based, self‐guided aerobic exercise programme. Participants aimed to progress to at least 150 min/week of walking or jogging at moderate intensity based on self‐perceived exertion reported on the Borg scale (rating of approximately 13) [20] by the end of the study. Participants who achieved 150 min/week of physical activity before the 12‐week interventional period ended were encouraged to continue increasing the duration of their weekly AT sessions, as long as self‐perceived exertion was not considered to be ‘hard’ or higher; no upper limit of physical activity was recommended by study investigators, and adherence was not recorded.

#### 2.3.4 COVID‐19‐Related Disruptions

**FIGURE 1:**
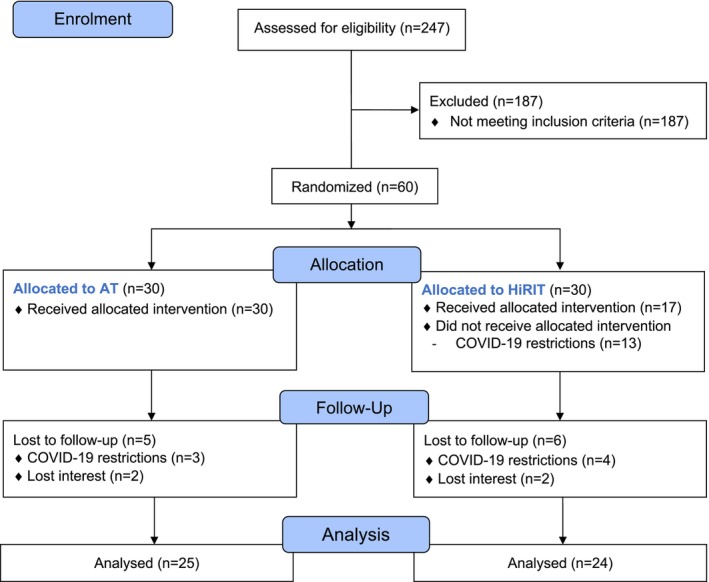
Study design and flow of participants.

During this trial, COVID‐19 lockdowns were implemented in Melbourne, Australia. The first lockdown commenced in March 2020, and over six more occurred throughout 2020 and 2021. These lockdowns prevented some participants from attending follow‐up appointments (Figure 1) and disrupted the HiRIT intervention for 13 participants to varying degrees. The high‐intensity programme could not be completed in the absence of barbell equipment in participant homes. Instead, HiRIT participants required to transition to periods of home‐based RT were instructed to complete the following exercises with weights or other heavy household items, if possible; calf raises, squats, wall or knee push‐ups and walking (20 min or more at a moderate intensity based on the Borg scale [20]).

### 2.4 Questionnaires and Anthropometry

Participants completed self‐administered questionnaires that collected information about general demographics and health (including the presence of chronic health conditions).

Height and weight were measured after an overnight fast (at least 8 hours) and participants were instructed to empty their pockets and remove heavy clothing and shoes. Weight was measured to the nearest 0.1 kg using electronic scales (Seca 804, Seca, Germany). Height was measured to the nearest 0.1 cm using a wall‐mounted stadiometer (Seca 213, Seca, Germany). BMI was calculated as weight in kilograms (kg) divided by height in metres squared (m^2^).

### 2.5 Body Composition

#### 2.5.1 DXA

Whole‐body dual‐energy x‐ray absorptiometry (DXA) (Hologic Discovery A, Hologic, USA) scans were performed to determine whole‐body fat, lean mass, body fat percentage and visceral adipose tissue area (VAT; cm^2^). Upper‐ and lower‐limb lean mass were summed to calculate appendicular lean mass (ALM). ALM corrected for height was calculated as ALM divided by height (m^2^). The manufacturer's spine phantom was used to calibrate the system on each scanning day, and the short‐term intra‐individual CVs for fat mass and ALM were 0.96% and 0.90%, respectively [21].

#### 2.5.2 pQCT

A single 2.5‐mm transverse scan (voxel size: 0.5 mm; scan speed: 20 mm/s) of the participant's non‐dominant leg was acquired using peripheral quantitative computed tomography (pQCT; Stratec XCT3000, Stratec Medizintechnik GmbH, Pforzheim, Germany). The length of the tibia was determined by measuring the distance between the tibial plateau and the distal tip of the medial malleolus. The scan site was located at 66% of the length of the tibia relative to the distal end. Locating scan sites was performed using a planar scout view of the distal tibia and placing a reference line parallel to the distal joint. An estimate of calf muscle density (mg/cm^3^) (tissue density within the muscle after removing bone and subcutaneous fat) was determined using the manufacturer's algorithms and software (Version 6.2). Scans were analysed using a smoothing filter (F03F05) and a threshold of 100 mg/cm^3^. The device was calibrated daily using the manufacturer's phantom, and the short‐term intra‐individual CV for muscle density was 0.97% [21].

#### 2.5.3 Liver Ultrasound

Patients completed a fasting ultrasound of their liver to assess steatosis. This test provided important structural information, such as the presence of biliary disease, and guided the staging of liver disease (if present). The test was performed at baseline and follow‐up by trained operators (Capitol Radiology, Victoria; data not presented).

### 2.6 Physical Function

#### 2.6.1 SPPB

Participants completed the SPPB, a validated tool for measuring physical performance and disability in older adults [22]. It consisted of three tasks; a chair stand test (also known as a five‐time sit‐to‐stand test), standing balance tests and a gait speed test. A score between 0 and 12 was calculated based on performance in these tests, and higher scores indicate better physical performance. Participants started the chair stand test from a standing position and were instructed to cross their arms touching opposite shoulders and attempt to sit and stand as quickly as possible five times without stopping, finishing in a seated position. Study staff started the clock as soon as they told participants to begin the test; they counted repetitions with participants and stopped the clock once the fifth repetition was completed (placed their back against the backrest). Tests were terminated if participants were unable to complete all five repetitions, used their arms for assistance, or the timer passed 1 min. Scores between 0 and 4 were noted based on the time taken to complete five stands. Standing balance tests included three variations with different difficulty: semi‐tandem, full‐tandem and side‐by‐side standing. When performing semi‐tandem stands, participants were instructed to place the heel of one foot alongside the big toe of the other foot and time in this position for 10 s. If completed successfully, participants were instructed to place their preferred foot directly in front of the other, touching heel‐to‐toes to complete a full‐tandem stand, and they were instructed to hold the position for 10 s. If participants could not hold the semi‐tandem stand for 10 s, they were instructed to stand with their feet touching side‐by‐side and hold this position for 10 s. Following completion of the balance tests, a score of 0–4 was assigned based on performance in these tests. Gait speed was measured using a 2.44‐m course, and a score of 0–4 was assigned based on the time it took to walk this distance at a normal walking speed.

#### 2.6.2 Hand Grip Strength

Hand grip strength was measured using a Jamar Plus Digital hydraulic hand grip dynamometer (Patterson Medical, Bolingbrook, IL, USA) [23]. In a seated position, participants were instructed to hold the instrument and flex their shoulder until their arm was parallel to the ground at shoulder height, grip and squeeze with the maximum force for 3–5 s. Participants were given 60 s of rest between each test, and once all tests were completed, the mean of the last two trials in the dominant hand was used to calculate hand grip strength (kg).

#### 2.6.3 Timed Up and Go Test

From a seated position with their backs positioned against the backrest, participants were instructed to stand up from a chair, walk to the end of a marked 3‐m‐long course at their usual walking speed, turn around, return to the chair and sit down [24]. The timer was started as a participant removed their back from the backrest and stopped once the participant had returned to the chair and placed their back against the backrest. The time taken to complete the trial was recorded.

#### 2.6.4 Stair Climb Test

Participants were instructed to climb a single flight of 10 steps as quickly as possible using the handrail for support if preferred [25]. After walking back to the bottom of the staircase, participants were given a 60‐s break. The test was repeated twice in total, and the average time taken to climb the flight of steps across both trials was used.

### 2.7 Objective Habitual Physical Activity Assessment

Participants were asked to wear an Actigraph wGT3X‐BT accelerometer (Actigraph, USA) at the hip for 1 week at baseline and 12 weeks to measure habitual physical activity levels. They were also provided with a diary to complete during each monitoring period to assist with interpreting the wear‐time validation performed using the Troiano algorithm in the manufacturer's software (ActiLife, Version 5.6.0). Participants were asked to report the time of day the device was put on and removed and note down any issues that may have influenced physical activity estimates (e.g. health issues resulting in low activity, activities that may have resulted in higher than usual physical activity estimates). Data were analysed in epoch lengths of 60 s. The average time spent performing moderate and vigorous physical activity (MVPA) per day and week was analysed using cut‐points proposed by Freedson and colleagues [26] (ActiLife, Version 5.6.0).

### 2.8 AEs

An AE was defined as any untoward medical occurrence in a participant during the study period, irrespective of whether it was related to study activities. Furthermore, a serious AE (SAE) was defined as any untoward medical occurrence that resulted in death, life‐threatening event, disability or incapacitation or required hospitalisation (minimum 1 overnight stay) at a hospital or emergency ward for observation and/or treatment that would not have otherwise been appropriate in a physician's office or outpatient treatment. Participants were provided with contact details of investigators and instructed to report any AE and/or SAE that occurred during the intervention period.

### 2.9 Sample Size Calculation

Change in gait speed from baseline to follow‐up was the primary study outcome. Previous studies have shown this to be a clinically meaningful predictor of falls, fractures and disability in older adults [27, 28] while not being limited by ceiling effects like the SPPB test [18]. A sample size of 60, with 30 participants allocated to each arm, provided > 80% power to detect a clinically meaningful 0.10 m/s (SD: 0.12 m/s) mean difference in gait speed between HiRIT and control groups, at an alpha level of 5% [28], after accounting for an expected 20% attrition rate.

### 2.10 Statistical Analysis

Normality assessments were performed for continuous data using boxplots and Shapiro–Wilk tests. Descriptive statistics were calculated as means ± standard deviation (SD), frequencies (percentages) or median (interquartile range [IQR]) for non‐parametric data. Our main analyses followed intention‐to‐treat principles and missing data were handled using linear mixed models. Per‐protocol analyses were also performed in participants who achieved at least 50% adherence to the HiRIT intervention (equivalent to one session per week); all AT participants were included in these analyses. Baseline and follow‐up values are presented as estimated marginal means with 95% confidence intervals. Twelve‐week changes in all outcome measures and group‐by‐time interactions were analysed using an unstructured covariance matrix. All models included the respective outcome measure as a dependent variable, time (2 levels), group (2 levels) and a group‐by‐time interaction term as fixed effects, with unique participant identifiers as a random intercept. For all analyses, a *p* value of < 0.05 or 95% confidence interval not including the null point was considered statistically significant. All data were analysed using SPSS Statistics Version 24 (IBM, USA).

## 3 Results

This cohort of older adults (mean age of 69.6 ± 6 years; 58% women) had a mean BMI of 32.9 ± 4.1 and SPPB score of 10.8 ± 1.1. The mean body fat percentage was 48.9 ± 3.7% in women and 38.9 ± 4.4% in men. Both groups had a similar mean age and proportions of women, smokers and participants with self‐reported hypertension (48%) (Table 1).

**TABLE 1:** Descriptive characteristics.

	AT (*n* = 30)	HiRIT (*n* = 30)
Age (years)	69.7 ± 5.5	69.5 ± 6.6
Women (%)	16 (53)	19 (63)
Smoker (%)		
Current smoker	0^a^	0^b^
Ex‐smoker	9 (33)^a^	14 (48)^b^
Never smoked	18 (67)^a^	15 (52)^b^
Self‐reported osteoporosis (%)	3 (10)^b^	1 (3)^b^
Self‐reported hypertension (%)	14 (48)	14 (48)

*Note:* Data are mean ± SD or frequency (%).

Abbreviations: AT, aerobic training; HiRIT, high‐intensity resistance and impact training.

^a^ *n* = 27.

^b^ *n* = 29.

Figure 1 presents the study design and flow of participants. We screened 247 individuals. Most were ineligible as they had SPPB scores > 11 or did not have obesity according to body fat percentage cut‐points. Sixty participants were randomised to either AT or HiRIT. All participants in the AT group received the allocated intervention; however, 13 participants in the HiRIT group had their gym programme interrupted to varying degrees and were required to perform low‐intensity home‐based RT and AT for different periods. Attrition was similar in both groups with reasons being COVID‐19 restrictions (HiRIT = 4; AT = 4) or loss of interest (both *n* = 2). HiRIT median gym session adherence was 62.5% (IQR: 31.3, 83.3) and affected by COVID‐19 restrictions. In HiRIT participants unaffected by COVID‐19 restrictions, median adherence was 81.3% (IQR: 71.9, 86.5). HiRIT participants who were affected by COVID‐19 lockdowns had a median adherence of 29.2% (IQR: 6.3, 47.9).

AEs in each group are presented in Table S1. There were no exercise‐related injuries reported in the AT group. In the HiRIT group, there was one exercise‐related injury (lower back strain), and this participant returned to the gym after a 10‐day break and completed the study without any ongoing issues. Two other injuries reported in the HiRIT group were possibly exercise‐related, specifically a minor muscle tear a participant noticed during recreational swimming and lower back strain noticed by another participant when they were moving houses; both participants continued exercising with one achieving 100% adherence despite the muscle tear. Both groups had participants who had lesions (kidney and pancreas), or previously unknown biochemical abnormalities (iron deficiency), detected during baseline appointments. In the AT group, one participant reported supplement‐related GI discomfort (unable to tolerate calcium and vitamin D tablets; the participant switched to a different brand with the same dose and did not report any issues), another participant had gastroenteritis, and one participant had a coronary event 1 month after completing the study, which was deemed to be a SAE unlikely to be related to the intervention.

**FIGURE 2:**
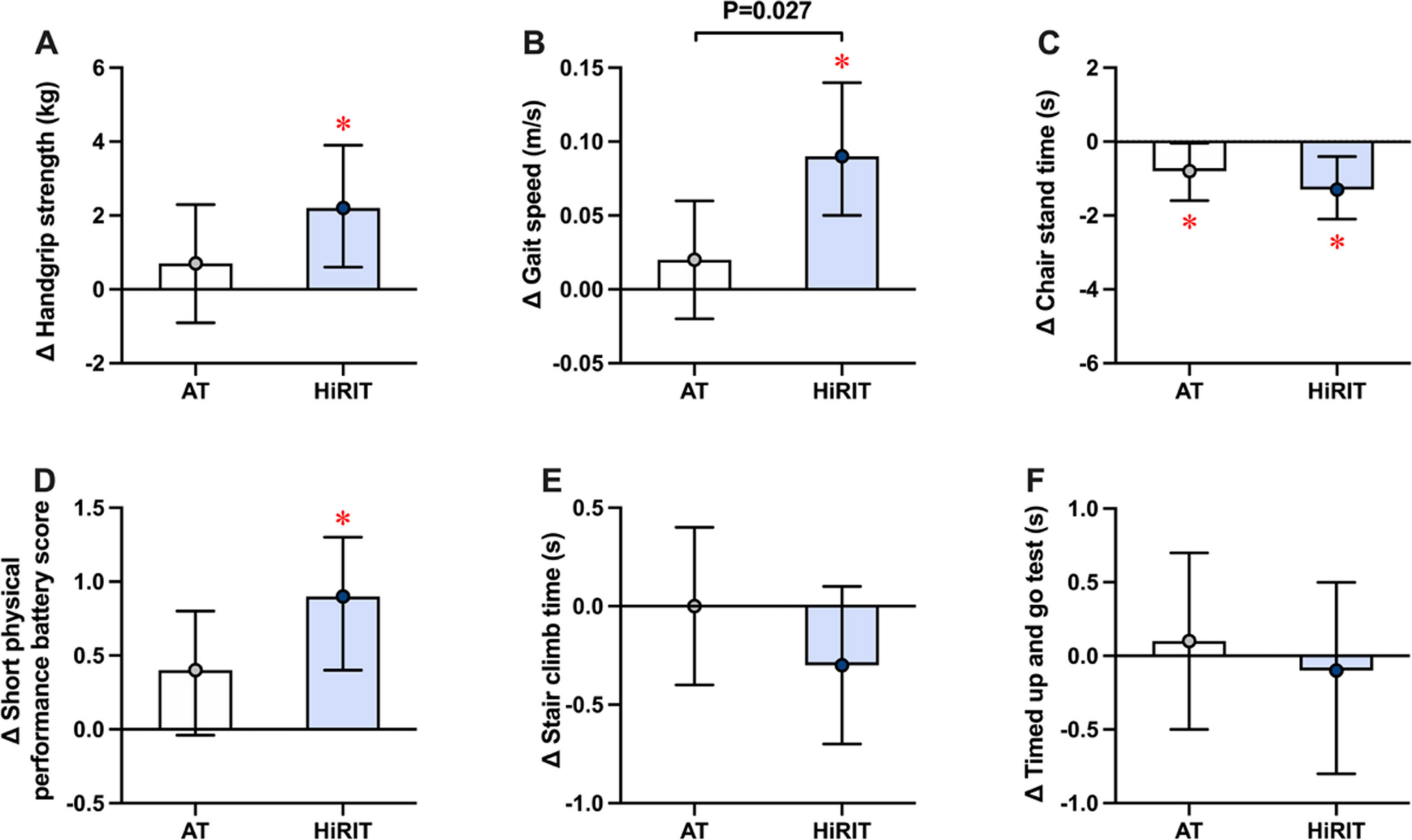
Twelve‐week mean changes (with 95% confidence intervals) in physical function outcomes following aerobic or high‐intensity resistance and impact training combined with weight loss. (A) Hand grip strength; (B) gait speed; (C) chair stand time; (D) short physical performance battery (SPPB) score; (E) stair climb time; (F) timed up and go test. AT, aerobic training; HiRIT, high‐intensity resistance and impact training; * denotes significant within‐group changes; P‐value represents significant between‐group difference.

**FIGURE 3:**
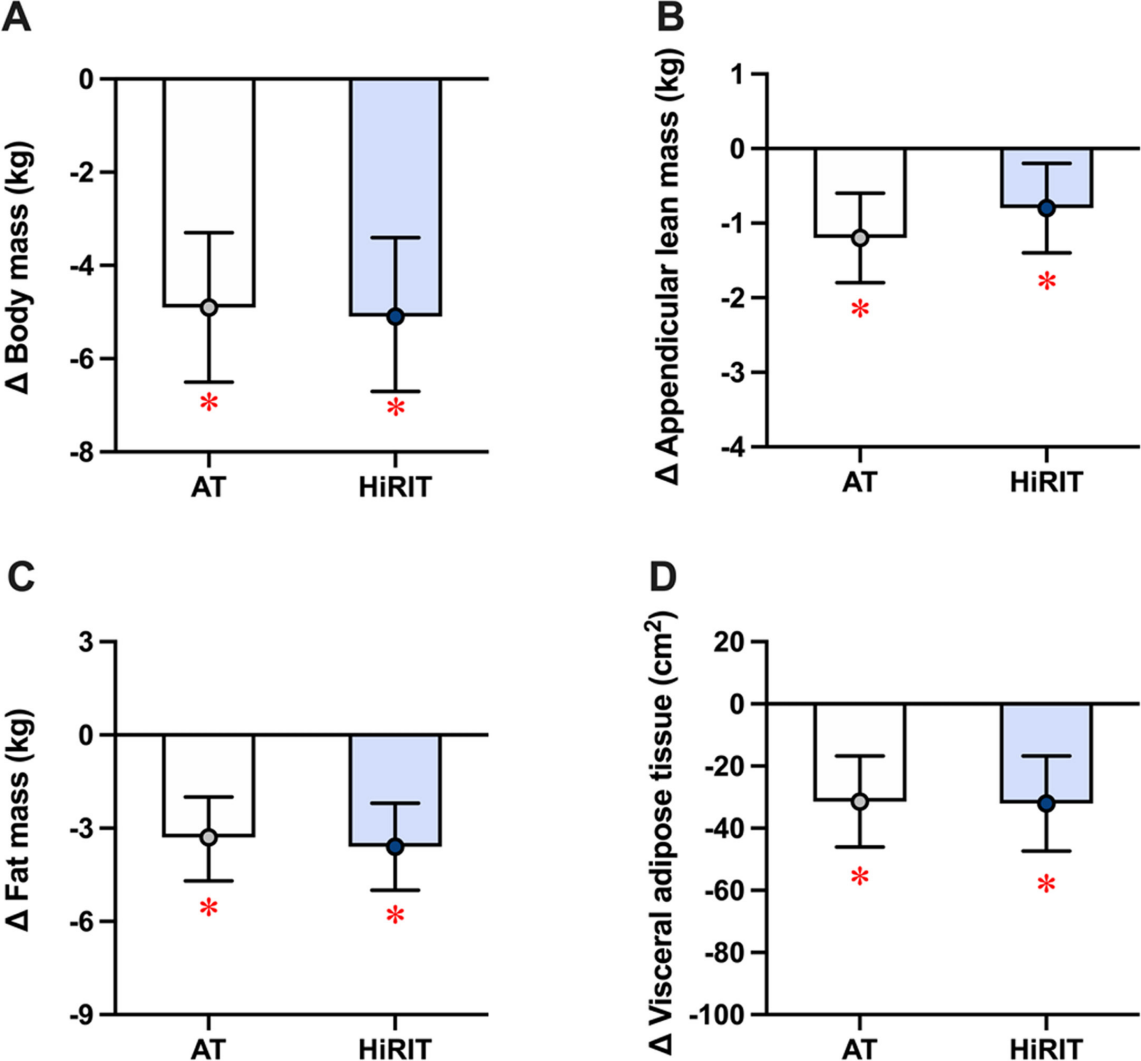
Twelve‐week mean changes (with 95% confidence intervals) in body composition outcomes following aerobic or high‐intensity resistance and impact training combined with weight loss. (A) body mass; (B) appendicular lean mass; (C) fat mass; (D) visceral adipose tissue area. AT, aerobic training; HiRIT, high‐intensity resistance and impact training; * denotes significant within‐group change.

Mean baseline and follow‐up values and intention to treat analyses comparing 12‐week changes in physical function and activity and body composition are presented in Figures 2 and 3 and Tables S2 and S3. Gait speed increased in the HiRIT group compared with the AT group. Both groups had within‐group decreases in chair stand times and HiRIT improved hand grip strength and SPPB scores; however, between‐group differences were not significant. Daily and weekly habitual MVPA levels were similar between groups and did not change throughout the intervention. Both groups had a reduction in body mass, BMI, ALM, fat mass, body fat percentage and visceral adipose tissue area. Per‐protocol analyses were performed on 46 participants (AT: 30; HiRIT: 18) who achieved at least 50% adherence to the HiRIT intervention (Tables S4 and S5). All results were unchanged.

## 4 Discussion

This 12‐week pilot RCT demonstrated that HiRIT improved gait speed compared with AT in older adults with obesity undertaking a dietary weight loss intervention. Other measures of physical function including hand grip strength and SPPB scores improved following HiRIT but not AT, although these changes did not differ between groups. Both groups had similar decreases in total body, fat and muscle mass and VAT. HiRIT proved to be both safe and feasible for this population, suggesting its potential as an effective exercise modality for older adults with obesity during weight loss.

HiRIT improved gait speed compared to AT, with the 0.07 m/s increase considered a small yet clinically meaningful change [28]. Distribution‐based estimates indicate that changes between 0.04 and 0.06 m/s are meaningful, whereas anchor‐based methods, which link changes to clinical benchmarks like self‐reported mobility improvement, provide similar estimates, with small meaningful changes ranging from 0.04 to 0.10 m/s [28]. These thresholds are based on datasets of different populations of older adults, underscoring their robustness and clinical relevance [28]. Other large cohort studies in older adults have shown that a 0.10 m/s higher gait speed is associated with 26% lower mobility disability risk, 18%–24% lower mortality risk (within 4 years) and a 7% reduction in fall risk [6, 29]. Several meta‐analyses have reported similar gait speed improvements following RT interventions [30, 31, 32, 33]. Surprisingly, our AT intervention involving progressive walking/jogging did not improve gait speed, despite this mode of exercise being ‘task‐specific’ and improving gait speed in other studies [34, 35]. Although participants in this study were asked to complete moderate‐intensity AT likely to elicit functional benefits, they were unsupervised, which is known to reduce intervention adherence [36] and might explain our findings. In a similar 6‐month RCT involving 160 older adults with obesity undergoing weight loss, RT produced comparable gait speed improvements to AT (RT: 9.3 m/min vs. AT: 8.1 m/min); however, exercise was supervised, and the AT group additionally completed flexibility and balance exercises, unlike the participants in our study [10].

Gait speed improvements following HiRIT may be explained by enhanced lower‐limb muscle function. Uematsu et al. reported that 8 weeks of light‐load high‐velocity leg‐press training in older adults improved usual and maximum gait speed, with increased hip extensor and ankle plantar flexor strength being the greatest contributors to these improvements [37]. We did not test for strength improvements in these specific muscle groups; however, they are targeted during squats and deadlifts (two compound exercises prescribed to HiRIT). Despite gait speed being the only measure of physical function to improve between groups, it should be noted that hand grip strength and SPPB scores improved following HiRIT but not AT. This pilot study may have been underpowered to detect between‐group differences in measures of physical function beyond gait speed.

Both groups had similar body composition changes and achieved clinically significant decreases in body mass (> 5%) [38]. Most of the body mass loss was attributed to decreases in fat mass, with a small amount resulting from lean mass losses. Although both groups lost similar amounts of lean mass, we expect that with a larger sample size and longer study duration, where the HiRIT intervention can be maintained (as opposed to temporarily replaced by a low‐intensity home‐based RT programme during COVID‐10 restrictions), we would have observed significant between‐group differences, with HiRIT attenuating weight loss‐related lean mass declines compared with AT. Several RCTs in similar populations have reported this finding [10, 11, 12]. Beyond lean mass preservation, completing RT or multimodal interventions involving RT augments weight loss‐related improvements in physical function and metabolic and bone health [10, 12, 39]. Further research is required to better understand what types of exercise are most effective for preserving musculoskeletal health during weight loss and to identify strategies that increase uptake of and adherence to these interventions in this population.

Adherence to the HiRIT intervention was high (81.3%) and attrition in both groups was low (7%) in participants who were unaffected by COVID‐19 restrictions. Adherence in this study was approximately 10% lower than in the LIFTMOR trial [15], but it is important to note all participants in this study completed a dietary weight loss intervention. Meta‐analyses have shown that adherence is approximately 60% [40] and attrition rates are as high as 28% [41] in studies prescribing non‐surgical weight loss interventions to adults with obesity. Interestingly, completing high‐intensity exercise did not increase attrition in the HiRIT group, which may have been related to the relatively low frequency and duration of weekly sessions (2 days; approximately 30 min). In support, a meta‐analysis of 55 studies showed that exercise intensity does not predict attrition, whereas longer session duration and higher weekly time effort and overall time effort per intervention, were associated with greater attrition in sedentary adults who participated in high‐intensity interval training interventions [42]. Similar to the LIFTMOR trial [15], we reported very few exercise‐related AEs, and a similar number to what would be expected in exercise trials [43], suggesting this intervention is safe in this population.

The findings of this study may have significant public health implications for older adults with obesity. The short‐term commercially available HiRIT programme we prescribed improved gait speed, a key indicator of mobility and predictor of adverse health outcomes [6, 29] and demonstrated safety and feasibility. These features underscore its potential to alleviate the healthcare burden associated with obesity and mobility impairments. Moreover, the high adherence rates and low attrition observed in the HiRIT group (unaffected by COVID‐19) suggest this intervention could be effectively implemented in community and clinical settings, where it may help preserve physical function and promote independence during weight loss. Future research should focus on assessing the scalability and cost‐effectiveness of this intervention, as well as its long‐term benefits on health outcomes.

Strengths of this study included a well‐characterised population of older adults with obesity (directly assessed using DXA) and a mobility limitation, a comprehensive battery of physical function and body composition outcome tests and data on important potential confounders such as habitual physical activity levels. Additionally, we successfully delivered an evidence‐based programme designed to improve bone density and physical function [15]. We also achieved high participant retention and adherence to the HiRIT training programme in participants unaffected by COVID‐19 restrictions.

Limitations of this study include COVID‐19‐related attrition, the absence of adherence data for the home‐based AT group and reduced training adherence in some HiRIT participants (*n* = 13) due to COVID‐19‐related intervention modifications. However, it is noteworthy that HiRIT still demonstrated improvements in gait speed compared with AT, despite the disruptions posed by the COVID‐19 pandemic. This underscores the robustness of the intervention and suggests outcomes could have been further enhanced under the originally planned study conditions. Analyses of all outcome measures except gait speed may have been underpowered, potentially increasing the risk of Type II errors and limiting the generalisability of our findings. Consequently, larger trials are required to confirm the effects of the study interventions on secondary outcomes and to improve the precision of treatment effect estimates. Although the duration of this study was sufficient to observe short‐term changes in physical function and body composition outcomes, it was not long enough to observe DXA‐estimated changes in bone outcomes, and a longer intervention would have likely led to greater treatment effects. We targeted individuals with SPPB scores below 11, so intervention‐related SPPB changes may have had limited sensitivity due to ceiling effects in our relatively well‐functioning population (mean baseline score in both groups was approximately 10.5) [18]. Our target population was composed only of older adults with obesity, meaning that although we included a population who would likely benefit from an exercise and weight loss intervention, our results might not be generalisable to similar populations with obesity and chronic conditions with contraindications to exercise, or more specifically, HiRIT. Finally, future studies could also be improved by including more accurate estimates of skeletal muscle mass, such as the D3‐creatine dilution method, which is more predictive of physical function, mobility and injurious falls compared with DXA‐determined lean mass [44].

In conclusion, this 12‐week pilot RCT demonstrated that HiRIT improves gait speed compared with AT in older adults with obesity undergoing a dietary weight loss intervention. HiRIT also improved other measures of physical function, including hand grip strength and SPPB scores, which were not observed following AT, although between‐group differences were not statistically significant. Both groups experienced clinically significant decreases in body mass (> 5%), and similar reductions in fat mass, lean mass and VAT. HiRIT was feasible and safe for this population, evidenced by high adherence, low attrition and very few exercise‐related AEs. Future trials with similar interventions, a larger sample size and longer durations (> 8 months) are warranted to determine whether HiRIT can attenuate weight loss‐related declines in muscle and bone mass.

## Conflicts of Interest

B.R.B. is the director of The Bone Clinic, Brisbane, Queensland, Australia. The other authors declare no conflicts of interest.

## Supporting information


**Table S1** List of related, potentially related and unrelated adverse events reported by study participants.
**Table S2.** Intention‐to‐treat analyses showing effects of aerobic or high‐intensity resistance and impact training combined with weight loss on physical function and habitual physical activity levels.
**Table S3.** Intention‐to‐treat analyses showing effects of aerobic or high‐intensity resistance and impact training combined with weight loss on body composition.
**Table S4.** Per‐protocol analyses showing effects of aerobic or high‐intensity resistance and impact training combined with weight loss on physical function.
**Table S5.** Per‐protocol analyses showing effects of aerobic or high‐intensity resistance and impact training combined with weight loss on body composition.

## Data Availability

The data that support the findings of this study are available from the corresponding author upon reasonable request.
