# Supplementary material for: Resistance and Impact Training During Weight Loss Improves Physical Function and Body Composition in Older Adults With Obesity
Source: J Cachexia Sarcopenia Muscle. 2025 Apr 9;16(2):e13789. doi: 10.1002/jcsm.13789 (PMC11981955; doi:10.1002/jcsm.13789)
Supplement: Supplementary file 1 — Table S1 List of related, potentially related and unrelated adverse events reported by study participants. Table S2. Intention‐to‐treat analyses showing effects of aerobic or high‐intensity resistance and impact training combined with weight loss on physical function and habitual physical activity levels. Table S3. Intention‐to‐treat analyses showing effects of aerobic or high‐intensity resistance and impact training combined with weight loss on body composition. Table S4. Per‐protocol analyses showing effects of aerobic or high‐intensity resistance and impact training combined with weight loss on physical function. Table S5. Per‐protocol analyses showing effects of aerobic or high‐intensity resistance and impact training combined with weight loss on body composition. [file JCSM-16-e13789-s001.docx]

**Supplementary Material**

**Table S1.** List of related, potentially related and unrelated adverse events reported by study participants.

|  | **AT** | **HiRIT** |
| --- | --- | --- |
| Injury (exercise-related) | 0 | 1 |
| Injury (possibly exercise-related) | 0 | 2 |
| Injury (not exercise-related) | 1 | 2 |
| Lesions detected in scans (not intervention-related) | 1 | 1 |
| Previously unknown biochemical abnormality (not intervention-related) | 1 | 1 |
| Supplement-related GI discomfort (intervention-related) | 1 | 0 |
| Gastroenteritis (not intervention-related) | 1 | 0 |
| Coronary event post-intervention (not intervention-related)* | 1 | 0 |

* – Serious adverse event; AT – aerobic training; HiRIT – high-intensity resistance and impact training.

**Table S2.** Intention-to-treat analyses showing effects of aerobic or high-intensity resistance and impact training combined with weight loss on physical function and habitual physical activity levels.

|  |  | **AT (N=25)** |  |  | **HiRIT (N=24)** |  |  | |
| --- | --- | --- | --- | --- | --- | --- | --- | --- |
|  | **Baseline** | **Follow-up** | **Within-Group Change** | **Baseline** | **Follow-up** | **Within-Group Change** | **Mean Difference** | **Group x Time (P-value)** |
| **MVPA**  **(min/week)** | 241.9  (181.4, 302.4) | 234.5  (158.8, 310.3) | -7.3  (-69.9, 55.3) | 191.9  (134.8, 249.1) | 193.7  (118.4, 269.1) | 1.8  (-62.0, 65.5) | 9.1  (-80.2, 98.4) | 0.838 |
| **MVPA**  **(min/day)** | 29.0  (21.6, 36.5) | 29.9  (20.4, 39.4) | 0.9  (-7.4, 9.2) | 24.8  (17.7, 31.8) | 26.0  (16.5, 35.5) | 1.3  (-7.2, 9.7) | 0.4  (-11.5, 12.2) | 0.951 |
| **Hand grip strength**  **(kg)** | 31.2  (27.0, 35.4) | 31.9  (27.7, 36.1) | 0.7  (-0.9, 2.3) | 27.9  (23.7, 32.0) | 30.1  (25.9, 34.3) | **2.2**  **(0.6, 3.9)** | 1.5  (-0.8, 3.8) | 0.199 |
| **Stair climb time**  **(s)** | 5.0  (4.5, 5.4) | 5.0  (4.6, 5.4) | 0.0  (-0.4, 0.4) | 5.1  (4.6, 5.6) | 4.8  (4.4, 5.3) | -0.3  (-0.7, 0.1) | -0.3  (-0.9, 0.3) | 0.289 |
| **Chair stand time**  **(s)** | 10.1  (9.3, 11.0) | 9.3  (8.4, 10.1) | **-0.8**  **(-1.6, -0.04)** | 10.1  (9.3, 11.0) | 8.8  (8.0, 9.7) | **-1.3**  **(-2.1, -0.4)** | -0.4  (-1.6, 0.7) | 0.457 |
| **Gait Speed**  **(m/s)** | 0.88  (0.84, 0.92) | 0.90  (0.85, 0.95) | 0.02  (-0.02, 0.06) | 0.87  (0.83, 0.91) | 0.96  (0.91, 1.01) | **0.09**  **(0.05, 0.14)** | **0.07**  **(0.01, 0.13)** | **0.027** |
| **SPPB score** | 10.5  (10.1, 10.8) | 10.9  (10.4, 11.3) | 0.4  (-0.04, 0.8) | 10.6  (10.2, 11.0) | 11.5  (11.0, 11.9) | **0.9**  **(0.4, 1.3)** | 0.5  (-0.1, 1.1) | 0.114 |
| **TUG time**  **(s)** | 8.3  (7.7, 8.8) | 8.3  (7.9, 8.8) | 0.1  (-0.5, 0.7) | 8.5  (7.9, 9.0) | 8.3  (7.9, 8.8) | -0.1  (-0.8, 0.5) | -0.2  (-1.1, 0.7) | 0.618 |

Data are mean (95% confidence intervals). Bold numbers indicate statistical significance (P<0.05). Mean differences are Δ HiRIT - Δ AT.

AT – aerobic training; HiRIT – high-intensity resistance and impact training; MVPA – moderate to vigorous physical activity; SPPB - short physical performance battery.

**Table S3.** Intention-to-treat analyses showing effects of aerobic or high-intensity resistance and impact training combined with weight loss on body composition.

|  |  | **AT (N=25)** |  |  | **HiRIT (N=24)** |  |  |  |
| --- | --- | --- | --- | --- | --- | --- | --- | --- |
|  | **Baseline** | **Follow-up** | **Within-Group Change** | **Baseline** | **Follow-up** | **Within-Group Change** | **Mean Difference** | **Group x Time (P-value)** |
| **Body mass**  **(kg)** | 89.4  (83.9, 94.8) | 84.4  (79.3, 89.6) | **-4.9**  **(-6.5, -3.3)** | 91.8  (86.3, 97.2) | 86.7  (81.5, 91.9) | **-5.1**  **(-6.7, -3.4)** | -0.2  (-2.5, 2.2) | 0.898 |
| **BMI**  **(kg/m^2^)** | 32.1  (30.6, 33.5) | 30.4  (29.0, 31.8) | **-1.7**  **(-2.3, -1.1)** | 33.6  (32.2, 35.1) | 31.7  (30.3, 33.1) | **-1.9**  **(-2.5, -1.3)** | -0.2  (-1.0, 0.6) | 0.646 |
| **ALM**  **(kg)** | 20.9  (19.0, 22.8) | 19.7  (18.0, 21.4) | **-1.2**  **(-1.8, -0.6)** | 19.9  (17.9, 21.8) | 19.1  (17.3, 20.9) | **-0.8**  **(-1.4, -0.2)** | 0.4  (-0.4, 1.3) | 0.288 |
| **ALM/Height^2^**  **(kg/m^2^)** | 7.4  (7.0, 7.9) | 7.0  (6.6, 7.4) | **-0.4**  **(-0.6, -0.2)** | 7.2  (6.7, 7.6) | 6.9  (6.5, 7.4) | **-0.3**  **(-0.5, -0.1)** | 0.1  (-0.1, 0.4) | 0.287 |
| **Lean mass**  **(kg)** | 48.9  (45.1, 52.6) | 47.3  (43.7, 50.9) | **-1.6**  **(-2.3, -0.8)** | 47.3  (43.4, 51.2) | 45.9  (42.1, 49.6) | **-1.4**  **(-2.2, -0.6)** | 0.2  (-0.9, 1.3) | 0.775 |
| **Fat mass**  **(kg)** | 36.8  (33.8, 39.8) | 33.5  (30.6, 36.4) | **-3.3**  **(-4.7, -2.0)** | 41.0  (38.0, 44.1) | 37.4  (34.4, 40.4) | **-3.6**  **(-5.0, -2.2)** | -0.3  (-2.2, 1.6) | 0.770 |
| **Body fat**  **(%)** | 43.2  (40.9, 45.4) | 41.5  (39.0, 44.1) | **-1.6**  **(-2.6, -0.7)** | 46.5  (44.1, 48.8) | 45.0  (42.4, 47.7) | **-1.4**  **(-2.4, -0.5)** | 0.2  (-1.2, 1.5) | 0.773 |
| **Visceral adipose tissue**  **(cm^2^)** | 223.4  (193.6, 253.2) | 191.9  (164.4, 219.4) | **-31.4**  **(-46.1, -16.8)** | 226.4  (195.6, 257.2) | 194.3  (165.8, 222.8) | **-32.1**  **(-47.4, -16.8)** | -0.7  (-21.8, 20.5) | 0.949 |
| **Muscle density**  **(mg/cm^3^)** | 69.8  (68.7, 70.9) | 70.0  (68.8, 71.1) | 0.2  (-0.6, 0.9) | 68.7  (67.7, 69.8) | 69.1  (68.1, 70.1) | 0.3  (-0.3, 0.9) | 0.2  (-0.9, 0.3) | 0.738 |

Data are mean (95% confidence intervals). Bold numbers indicate statistical significance (P<0.05). Mean differences are Δ HiRIT - Δ AT.

AT – aerobic training; HiRIT – high-intensity resistance and impact training; BMI – body mass index; ALM – appendicular lean mass.

**Table S4.** Per protocol analyses showing effects of aerobic or high-intensity resistance and impact training combined with weight loss on physical function.

|  |  | **AT (N=25)** |  |  | **HiRIT (N=18)** |  |  | |
| --- | --- | --- | --- | --- | --- | --- | --- | --- |
|  | **Baseline** | **Follow-up** | **Within-Group Change** | **Baseline** | **Follow-up** | **Within-Group Change** | **Mean Difference** | **Group x Time (P-value)** |
| **Hand grip strength (kg)** | 31.2  (26.7, 35.7) | 31.9  (27.4, 36.4) | 0.7  (-1.0, 2.4) | 28.1  (22.3, 33.9) | 30.8  (25.1, 36.5) | **2.6**  **(0.6, 4.7)** | 1.9  (-0.8, 4.6) | 0.156 |
| **Stair climb time**  **(s)** | 5.0  (4.5, 5.4) | 5.0  (4.5, 5.4) | 0.0  (-0.4, 0.4) | 5.5  (4.9, 6.1) | 5.1  (4.6, 5.6) | -0.4  (-0.9, 0.1) | -0.4  (-1.1, 0.3) | 0.217 |
| **Chair stand time**  **(s)** | 10.1  (9.3, 10.9) | 9.3  (8.4, 10.2) | **-0.8**  **(-1.7, -0.03)** | 10.3  (9.2, 11.3) | 8.9  (7.8, 10.0) | **-1.4**  **(-2.4, -0.4)** | -0.5  (-1.8, 0.8) | 0.416 |
| **Gait Speed**  **(m/s)** | 0.88  (0.84, 0.92) | 0.90  (0.85, 0.95) | 0.02  (-0.02, 0.06) | 0.87  (0.81, 0.92) | 0.96  (0.9, 1.0) | **0.09**  **(0.04, 0.15)** | **0.07**  **(0.01, 0.1)** | **0.037** |
| **SPPB score** | 10.5  (10.1, 10.9) | 10.9  (10.5, 11.3) | 0.4  (-0.05, 0.8) | 10.6  (10.0, 11.1) | 11.5  (11.1, 12.0) | **1.0**  **(0.5, 1.5)** | 0.6  (-0.1, 1.3) | 0.084 |
| **TUG time**  **(s)** | 8.3  (7.7, 8.8) | 8.3  (7.9, 8.8) | 0.08  (-0.5, 0.7) | 8.6  (7.8, 9.3) | 8.6  (8.0, 9.2) | 0.04  (-0.8, 0.9) | -0.04  (-1.1, 1.0) | 0.940 |

Data are mean (95% confidence intervals). Bold numbers indicate statistical significance (P<0.05). Mean differences are Δ HiRIT - Δ AT.

AT – aerobic training; HiRIT – high-intensity resistance and impact training; SPPB - short physical performance battery.

**Table S5.** Per protocol analyses showing effects of aerobic or high-intensity resistance and impact training combined with weight loss on body composition.

|  |  | **AT (N=25)** |  |  | **HiRIT (N=18)** |  |  | |
| --- | --- | --- | --- | --- | --- | --- | --- | --- |
|  | **Baseline** | **Follow-up** | **Within-Group Change** | **Baseline** | **Follow-up** | **Within-Group Change** | **Mean Difference** | **Group x Time**  **(P-value)** |
| **Body mass**  **(kg)** | 89.4  (83.5, 95.2) | 84.4  (79.0, 89.9) | **-4.9**  **(-6.6, -3.2)** | 92.7  (85.2, 100.2) | 87.5  (80.5, 94.5) | **-5.2**  **(-7.2, -3.1)** | -0.3  (-3.0, 2.4) | 0.847 |
| **BMI**  **(kg/m^2^)** | 32.1  (30.5, 33.7) | 30.4  (28.9, 31.9) | **-1.7**  **(-2.3, -1.1)** | 33.7  (31.6, 35.7) | 31.7  (29.8, 33.7) | **-1.9**  **(-2.7, -1.2)** | -0.2  (-1.2, 0.7) | 0.629 |
| **ALM**  **(kg)** | 20.9  (18.9, 22.8) | 19.7  (17.9, 21.4) | **-1.2**  **(-1.8, -0.6)** | 19.8  (17.3, 22.3) | 18.7  (16.5, 20.9) | **-1.1**  **(-1.8, -0.4)** | 0.12  (-0.8, 1.1) | 0.790 |
| **ALM/Height^2^**  **(kg/m^2^)** | 7.4  (7.0, 7.9) | 7.0  (6.6, 7.4) | **-0.4**  **(-0.6, -0.2)** | 7.1  (6.5, 7.7) | 6.7  (6.2, 7.3) | **-0.4**  **(-0.6, -0.1)** | 0.0  (-0.3, 0.4) | 0.769 |
| **Lean mass**  **(kg)** | 48.9  (45.0, 52.8) | 47.3  (43.5, 51.0) | **-1.6**  **(-2.4, -0.8)** | 47.0  (42.0, 52.1) | 45.7  (40.8, 50.5) | **-1.4**  **(-2.4, -0.4)** | 0.2  (-1.1, 1.5) | 0.770 |
| **Fat mass**  **(kg)** | 36.8  (33.8, 39.8) | 33.5  (30.5, 36.4) | **-3.3**  **(-4.7, -2.0)** | 41.5  (37.6, 45.4) | 38.2  (34.4, 42) | **-3.3**  **(-5.0, -1.6)** | 0.0  (-2.1, 2.2) | 0.982 |
| **Body fat**  **(%)** | 43.2  (40.9, 45.5) | 41.5  (39.0, 44.1) | **-1.6**  **(-2.6, -0.7)** | 46.8  (43.9, 49.8) | 45.6  (42.3, 48.9) | **-1.2**  **(-2.4, -0.1)** | 0.4  (-1.1, 1.9) | 0.609 |
| **Visceral adipose tissue**  **(cm^2^)** | 223.4  (193.6, 253.1) | 191.9  (164.0, 219.8) | **-31.4**  **(-46.7, -16.2)** | 226.5  (188.0, 264.9) | 194.7  (159.2, 230.3) | **-31.7**  **(-50.4, -13.1)** | -0.3  (-24.4, 23.8) | 0.981 |

Data are mean (95% confidence intervals). Bold numbers indicate statistical significance (P<0.05). Mean differences are Δ HiRIT - Δ AT.

AT – aerobic training; HiRIT – high-intensity resistance and impact training; BMI – body mass index; ALM – appendicular lean mass.
